# Enzymatic production of glucosamine and chitooligosaccharides using newly isolated exo-β-d-glucosaminidase having transglycosylation activity

**DOI:** 10.1007/s13205-015-0330-5

**Published:** 2016-01-06

**Authors:** Sujata Sinha, Subhash Chand, Pushplata Tripathi

**Affiliations:** 1 Department of Biochemical Engineering and Biotechnology, Indian Institute of Technology, Delhi, 110016 India; 2 School of Sciences, Indira Gandhi National Open University, Maidan Garhi, New Delhi, 68 India

**Keywords:** Glucosamine, Immobilization, Transglycosylation, Chitosanase

## Abstract

Exochitosanase secreting fungus (*A*. *fumigatus* IIT-004) was isolated from fish waste using 1 % (w/v) chitosan as sole carbon source after multistage screening. Chitosan-dependent exochitosanase enzyme production (6 IU ml^−1^) in log phase of growth (chitosan utilization rate 0.11 g g^−1^ cell h^−1^) was observed for *Aspergillus fumigatus* in chitosan minimal salt medium and there was no enzyme production in glucose medium. Enzyme production was found to be extracellular and subjected to purification by a number of steps like acetone fractionation as well as column chromatography. 40 % yield and 26-fold of enzyme purification was achieved after all the steps. Purified enzyme was characterized for optimum temperature, pH, ionic strength and substrate specificity. The *K*_m_ and *V*_max_ for purified exochitosanase enzyme was calculated to be 8 mg ml^−1^ and 5.2 × 10^−6^ mol mg^−1^ min^−1^. Enzyme was immobilized on polyacrylonitrile nanofibres membrane matrix by adsorption as well as amidination. Enzymatic production of glucosamine was achieved using various chitosan substrates by free/immobilized exochitosanase and compared. Isolated and purified exochitosanase also showed transglycosylation activity.

## Introduction

Chitosanase are enzymes which specifically hydrolyze chitosan to produce chitooligomers and monomers, i.e. glucosamine and *N*-acetyl-d-glucosamine, which depends on types of enzyme. On the basis of their mode of action, two types of chitosanase enzymes have been reported: exo-(EC 3.2.1.165) and endochitosanase (EC 3.2.1.132). Endo-acting enzymes hydrolyse β-1,4 linkages between Gln (glucosamine) residues in partly acetylated chitosan. It is used for production of chitooligosaccharides (COS) by acting on reducing end of chitosan; however, exo-enzymes attacks chitosan from non-reducing end of COS or chitosan to produce Gln (glucosamine) or *N*-acetyl-d-glucosamine residue (NAG) (Thadathil and Velappan 2014). Endo- and exochitosanase chitosanases have been reported from a number of bacteria, fungi, actinomycetes and plants (Somashekar and Joseph 1996); however, the study of endochitosanases is more frequent than exo-acting enzymes. Frequent reports of endo-acting chitosanase enzymes can be attributed to the increasing importance of COS in various fields. Importance of study of exochitosanases has been undermined comparatively.

The end products of exochitosanase hydrolysis are Gln and NAG, which are known for their therapeutic value and used as food supplements. NAG and Gln are used for the treatment of osteoarthritis; however, hydrochloride and sulfate salts of glucosamine are bitter in taste and are avoided. NAG is sweet in taste and is preferred over Gln to be administered orally when used as food supplement, as the therapeutic activity of both the molecule is similar (Kajimoto et al. 1998). NAG and Gln are produced by acid hydrolysis of chitin/chitosan; however, this is not considered to be natural and leads to lot of toxic waste with lower yield (Sakai 1995).

Very few studies on enzymatic production of Gln and NAG have been reported in literature. Exochitosanase enzyme can be used for production of these molecules in an eco-friendly way from various chitosan substrates as they are able to release Gln and NAG from non reducing end of chitosan/chitin molecule.

A new exochitosanase enzyme was isolated from *Aspergillus fumigatus* IIT-004, which also showed transglycosylation activity. Enzyme was immobilized on PANNFM and was used for hydrolysis of chitosan. Isolated enzyme was also purified, characterized, immobilized and was used for chitodimer production using transglycosylation activity.

## Materials and methods

### Materials

Commercial chitosan from crab shell, average molecular weight 290 kDa, 93 % *N*-deacetylated (DAC); were kind gift from Marine chemicals, Chennai, India. Chitosan from shrimp shell (>75 % deacetylated) were procured from Sigma-Aldrich, Germany. Glucosamine HCl and Chitin were purchased from Hi-media. All other chemicals were procured from SRL, Mumbai, India and were of analytical grade. For isolation and screening, soil was taken from fish market, Chittaranjan Park, New Delhi, India.

### Isolation and screening

Chitosan/colloidal chitosan minimal salt (CMS/CCMS) medium (1 % w/v) was used for isolation and screening of chitosanase producing microbes from soil. Soil samples were weighed, suspended in sterile water and plated after serial dilutions (10^−1^ to 10^−6^). Composition of the medium was as follows: 0.5 % (w/v) chitosan, 0.5 % yeast extract, 0.2 % K_2_HPO_4_, 0.1 % KH_2_PO_4_, 0.07 % MgSO_4_·7H_2_O, 0.05 % NaCl, 0.05 % KCl, 0.01 % CaCl_2_ and 2 % Bacto Agar with final pH 6.8.  %. In case of CCMS agar medium 90 ml of colloidal chitosan was added in place of chitosan. The pH was adjusted to 6.0–7.0 before autoclaving. Colloidal chitosan was prepared by dissolving 15 g of 90 % deacetylated chitosan in 1 l (Yabuki et al. 1987).

Microbial colonies grown on agar plates were point inoculated to CMS broth (100 ml) for screening. Samples were withdrawn at regular interval (24 h) till 1 week and biomass was removed by centrifugation at 4500×*g* for 20 min at 4 °C. Chitosanase assay was done by dinitrosalicylic (DNS) acid method in supernatant collected after centrifugation of samples. Microbial isolates showing significant chitosanase enzyme activity (≥4 U ml^−1^) were taken for second stage of screening. Selected microbes after first stage of screening were again inoculated in 100 ml of CMS broth and were grown at room temperature on rotary shaker (180 rpm) for 1 week. 1 ml of samples was withdrawn at 16 h time interval and biomass was removed by centrifugation. Exo-/endochitosanase enzyme activity was checked in supernatant by reducing sugar estimation (DNS method) and thin layer chromatography (TLC).

In the third stage of screening, inducible/constitutive and specific activity of secreted enzyme was checked. Constitutive production was checked by growing isolates in the 100 ml medium containing glucosamine/glucose (1 % w/v) as carbon source and yeast extract (0.2 % w/v) as nitrogen source along with minimal salts (same as in CMS).

In the fourth stage of screening, the axenic cultures of the selected microorganisms were inoculated in 200 ml of chitosan minimal salt broth medium in 1 l of Erhlenmeyer flask (Borosil, India) and incubated at 28 °C on orbital shaker at 250 rpm for one week. 5 ml of samples was withdrawn at 8 h interval and cells were harvested by centrifugation (5500×*g* for 20 min at 4 °C) in pre-weighed falcon tubes or filtration in case of mycelia. The supernatant was checked for extracellular chitosanase enzyme activity. Protein estimation was done by Bradford’s assay and change in medium pH was also monitored with time. Biomass estimation was done by taking dry cell weight for fungal culture and OD 600 nm was recorded for bacterial culture.

#### Preparation of chitosan and enzyme assay

Chitosanase activity was determined by measuring the reducing sugars produced from chitosan. 300 µL of the crude enzyme was mixed with 500 µL of chitosan (1 % w/v) and 700 µL of acetate buffer (200 Mm, 5.5). Reaction mixture was incubated for 30 min at 37 °C. Enzyme was deactivated by heating the reaction mixture at 100 °C for 2 min. The reducing sugars in the supernatant were measured by using modification of Schales (1945) method using glucosamine HCl as the calibration standard. One unit of chitosanase was defined as the amount of enzyme that liberated 1 μmol of glucosamine per min under the standard conditions.

### Enzyme purification

Enzyme was purified from culture fluid of *Aspergillus* after growing for 96 h in a flask containing chitosan minimal salt broth. Production was achieved by inoculation of 1 % (v/v) of spore suspension (4 × 10^5^ spores ml^−1^) in 2.0 L Erhlenmeyer flasks containing 0.5 l production medium and was incubated on an orbital shaker (200 rpm) at 32 °C. Biomass was harvested by mycelium filtration and all purification steps were performed at 4 °C. Chitosanase activity was checked in the supernatant collected after centrifugation. Acetone fractionation was done by adding four times the volume of chilled Acetone to 0.5 l volume of the culture broth at 4 °C with continuous stirring. Mixture was left for 4 and 12 h and precipitated protein was recovered after centrifugation at 6000×*g* for 30 min. Pellet was collected and was left for acetone evaporation at room temperature. After complete evaporation of acetone, pellet was suspended in minimum volume of sodium acetate buffer (100 mM, 5.5 pH) till further use at 4 °C. Chitosanase assay was done by DNS and protein estimation was done by Bradford’s method in the suspension and was compared to crude chitosanase enzymes activity in culture broth. Protein was loaded on pre equilibrated DEAE Sepharose column (1 × 20 CM, Merck) with acetate buffer. A flow rate of 2 ml min^−1^ was maintained and 5 ml fractions were collected. Unbound proteins were washed with buffer and enzyme protein was eluted with 0.1–1 M NaCl in Tris–HCl buffer (pH 7.0) gradient. Active fractions were loaded on CM-Sepahrose, bound protein from which were eluted by applying linear 1000 ml gradient of 0.1–1.0 M NaCl in Tris–HCl buffer (pH 7.0). Chitosanase enzyme assay was done in each fraction pooled from the columns and was concentrated. Concentrated fractions were loaded onto fast flow Superdex™ (Pharmacia, FPLC) 200 columns (2 × 20 cm) GL and 5 ml of active fraction were collected after maintaining a flow rate of 1.8 ml min^−1^. Purified chitosanase enzyme was stored at 4 °C.

### Enzyme characterization

The kinetic parameters were determined by measuring the initial rates of chitosan degradation as a function of chitosan concentration in the range of 10–200 mM (>90 % DAC, 290 kDa) under standard reaction conditions at 37 °C. Reaction rates were measured by monitoring the release of glucosamine upon hydrolysis of chitosan at 540 nm. *V*_max_ and *K*_m_ were determined by least- squares regression analysis of initial velocity versus chitosan concentration using the double-reciprocal plots of Line weaver- Burk transformation. Discontinuous SDS-PAGE (10 %) was carried out to determine apparent molecular mass. Protein assay was done by Bradford’s method (Bradford 1976). Isoelectric point was determined by method of Su et al. (2006). Acetate buffer was used for pH range 3.5–5.5, phosphate buffer, 6.5–7.5, and Tris–HCl was used for pH, 8.0 and above for determination of pH optimum. Enzymatic reactions were carried out in respective buffers with 1 % w/v chitosan as substrates during enzyme assay; pH stability was checked by incubating enzymes in respective buffers (3.0–9.0) with an interval of 1.0 unit pH for 10 min and doing enzyme assays. Optimum temperature was determined by carrying out enzymatic reaction at various temperatures (20–100 °C) with an interval of 10 °C using 1 % w/v chitosan as substrates in sodium acetate buffer (200 Mm, 5.5). Thermal stability of enzyme was checked by incubating enzyme solution in acetate buffer at these temperatures from 10 to 80 min. Samples were withdrawn at an interval of 10 min and assay was done with 1 % w/v chitosan as substrate at 37 °C. To determine the effect of metal ions like Ba^2+^, Ca^2+^, CO^2+^, Cu^2+^, Fe^2+^, Hg^2+^, Mn^2+^, Ni^2+^ or Zn^2+^ was added in form of chloride salt to the enzyme solution (5 ml) at a final concentration of 10 mM. Ethylenediaminetetraacetic acid (EDTA), *N*-bromosuccinimide (NBS), monoiodeacetic acid (IAA), *N*-ethylmaleimide (NEM), *p*-chloromercuribenzoate (pCMB), Tryptophan modifier 2-hydroxy-5-nitro benzyl bromide (HNB), Tween-20, and Triton X-100 were added to enzyme solution at final concentration of 1 mM to determine the effect of organic substances. The enzyme activity was assayed after incubating at 37 °C for 30 min. Various types of chitosan substrates with degree of deacetylation (DAC) ranging from 50 to 90 %, colloidal chitosan, chitin, colloidal chitin, cellulose, starch were used for enzyme assay to check its substrate specificity.

### Transglycosylation

Various concentrations (5, 10, 15 and 20 % w/v) of ammonium sulfate, sodium sulphate and sodium chloride were checked for inducing transglycosylation activity. Concentration of Gln and NAG were varied from 0.1 to 5 g/l in the reaction mixture. Final composition of reaction mixture after optimization was as follows: 100 mg of Gln and 500 mg of NAG were mixed in 100 ml of acetate Buffer (200 mM, 5.5) containing 20 % of ammonium sulfate. 2 ml of enzyme (6 U ml^−1^) was added to this mixture and incubated till 48 h at 50 °C temperature. Reaction mixture became turbid with time and eventually a precipitate was formed, which was collected after centrifugation. Precipitate was washed with 5 ml of aqueous methanol (20 %) solution and was dissolved in 10 ml of water. Insoluble component was removed by centrifugation (5000×*g* for 10 min). Supernatant was collected and chitooligomers presence was checked by thin layer chromatography (TLC). Supernatant containing COS was lyophilized and stored at 4 °C.

### Enzyme immobilization

Enzyme was immobilized on electrospun polyacrylonitrile nanofibrous membranes (PANNFM) matrix and reaction conditions were optimized for maximum protein binding. PANNFM (average diameter 200 nm), a kind gift from National Physical Laboratory, New Delhi, India were used for enzyme immobilization by amidination reaction. Nanofibres (0.5 g) surface was treated with absolute ethanol (100 ml) and was bubbled with 1 N HCl (2 ml) for 30 min to produce the corresponding imidoester derivatives, washed with acetate buffer and treated with enzyme (6 U ml^−1^, 1–10 mg ml^−1^) solution. Mixture was shaken at room temperature for 6 h and again washed with acetate buffer (200 mM, 5.5 pH) several times to remove unbound enzymes. NFM bound enzymes were lyophilized and were stored for further use. Protein assay was done in supernatant and bound enzyme activity was checked by 3, 5-dinitrosalicylic acid (DNS) method by measuring the rate of release of reducing sugar. 0.1 g of PANNFM bound enzymes were added to 3 ml of 1 % chitosan solution (90 % DAC, 290 KDa) and incubated at 37 °C for 30 min in a water bath. The reaction was stopped by adding 0.5 ml of 1 N NaOH solution and sample was withdrawn for enzyme and protein assay [6]. The withdrawn reaction mixture was centrifuged at 4500×*g* for 15 min to remove the chitosan and the concentration of reducing sugar was determined in supernatant. One unit of enzyme activity was defined as the amount of enzyme that could produce 1 µmol reducing sugar (glucosamine) per min.

Immobilization efficiency for chitosanase enzyme was calculated by the following formula:

$${\text{Specific}}\;{\text{activity}}\;{\text{of}}\;{\text{immobilized}}\;{\text{enzymes}}\left( {{\text{U}}\;{\text{mg}}^{ - 1} } \right)/{\text{specific}}\;{\text{activity}}\;{\text{of}}\;{\text{soluble}}\;{\text{chitosanase}}\;{\text{enzyme }}\left( {\text{U/mg}} \right) \, \times100.$$

Thermal activity and stability of free and immobilized enzymes were measured over a temperature range of 20–90 °C with an interval of 10 °C. PANNFM-chitosanase was incubated in acetate (200 mM, 5.5 pH) buffer at temperatures of 10–90 °C for 30 min and was added to mixture containing acetate buffer (200 mM, 5.5 pH) and chitosan (1 % W/V) was used as substrate (5 ml) for enzyme assay. Release of reducing sugar by incubated PANNFM immobilized enzymes were compared with that of lyophilized enzymes as control. Buffers like glycine–HCl (2.2–3.6); sodium acetate (3.6–5.6); sodium phosphate (5.8–8.5) and glycine-NaOH (8.6–10.6) buffers were used to check stability and activity of PANFNM-chitosanase at different pH. Enzymes were incubated in the above-mentioned buffers suitable in different pH ranges and residual activity was checked for stability studies. Optimum pH for chitosanase enzymes activity was checked by carrying out the enzymatic reactions in these buffers at 37 °C for 30 min. Reusability were evaluated by washing the membrane with acetate buffer (200 mM, 5.5 pH) after completion of each reaction cycle. Chitosan (1 % w/v), 3 ml was used as substrate for starting each new cycle of reaction and this process was repeated up to ten cycles. Storage stability was checked by storing in acetate buffer at 4 °C. Activity was checked after every five days and was monitored up to 60 days. TLC and High-performance TLC (HPTLC) were used for qualitative and quantitative estimation of COS. Hydrolysis of chitosan was done by adding 0.5 g of PANNFM-chitosanase to 50 ml of 1 % (w/v) of chitosan in 200 mM acetate buffer (pH 5.5) at 37 °C temperatures up to 24 h. Samples were withdrawn at each 1 h time interval and absolute ethanol was added to precipitate COS. Insoluble chitosan was removed by centrifugation (3000×*g* for 10 min) and supernatant was analyzed for COS production. Samples were concentrated by rotary vacuum evaporation and were subjected to TLC and HPTLC on a silica gel plate (Merck 60, GF-254).

### Glucosamine and COS analysis

Gln production using free and immobilized enzyme was carried out in batch condition. Reaction mixture of 1 l was taken in 3.7 l bioreactor at 40 °C and was kept under constant stirring condition. Composition of reaction mixture was in ratio of 4:3:3 (sodium acetate buffer: chitosan (1 % w/v): enzyme: 11 U/mg). 1 g of PANNFM bound enzyme was taken in place of soluble enzyme. Hydrolysis with enzymes was continued till 3 h and aliquots were periodically withdrawn (10 min) from the reaction mixture and heated at 100 °C for 2 min for stopping the enzymatic reaction. Reaction mixture was filtered through whatman filter paper, concentrated by rotary vacuum evaporator at 60 °C and was precipitated with five volumes of ethanol. The precipitate was dried at 60 °C for 2 h and assay was done by DNS method in all the cases of study. Yield of glucosamine production by soluble and immobilized enzyme was calculated by following formula and compared:

$${\text{g}}\;{\text{of}}\;{\text{glucosamine}}\;{\text{produced}}/{\text{g}}\;{\text{of}}\;{\text{chitosan}} \times 100$$

COS was precipitated after addition of absolute ethanol (2 ml) in the supernatant. Samples were concentrated by rotary vacuum evaporation and 10 µl volumes were subjected to TLC on a silica gel plate (Merck 60, GF-254). Solvent system composed of 1-propanol:water:concentrated ammonia (7:2:1) was used. The plate was stained using 0.1 % ninhydrin dissolved in ethanol and plates were visualized by drying the plates in an oven at 100 °C for 20 min. COS standards were spotted on TLC plates and were run in the parallel to the sample.

## Results

### Isolation and screening

Out of 80 colonies grown on CMS/CCMS agar medium, 23 were selected for screening on the basis of morphological diversity. Microbial colony morphology was more diverse on CMS agar medium (Hsiao et al. 2008) as compared to CCMS agar medium (Su et al. 2006). Enzyme activity ranged from 1 to 6 U ml^−1^ and six microorganisms were taken for second stage of screening had equal or more than 4 U ml^−1^ of crude chitosanase activity. None of the isolates showed endochitosanase activity and four highest exo-enzyme producer (4.0, 6.0, 5.2 and 6.0 U ml^−1^) were checked for their specific activity.

All the four strains taken for third stage of screening were found to be secreting chitosanase extracellular and production which was induced by chitosan in all the four microbial strains. Two of them were selected after third stage screening on the basis of highest specific activity (0.7 and 0.4 U mg^−1^). Submerged fermentation studies in CMS broth were carried out with two isolates for one week and enzyme production, specific activity, pH and growth (dry cell weight) was recorded with time of growth. On the basis of least time, more growth, chitosanase enzyme-specific activity and pH, FM-2 was selected for exochitosanase enzyme production and detail study.

### Culture growth kinetics and enzyme production in submerged fermentation

**Fig. 1:**
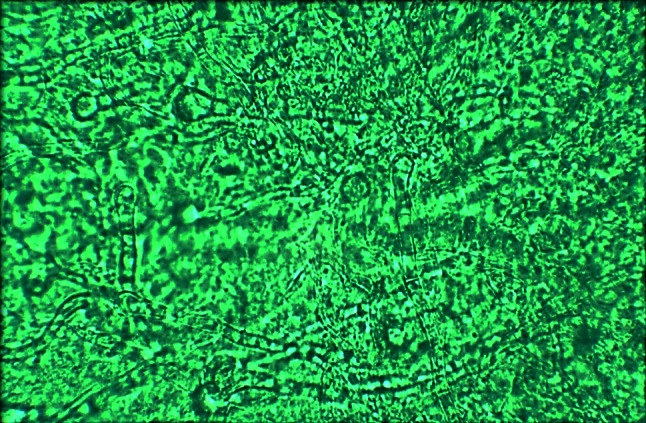
Photomicrograph of mycelium of *Aspergillus* *fumigatus* IIT-004 (×100)

Identification and characterization of enzymes producing isolates were done morphologically, physiologically as well as based on genetic homology studies. The fungal strain showing exochitosanse enzyme production was identified as *Aspergillus fumigatus* (IIT-004) by 18S rDNA gene homology. Sequences for this new isolate producing exochitosanase have been submitted in the Gen Bank (ID: JF708947) (Fig. 1).

**Fig. 2:**
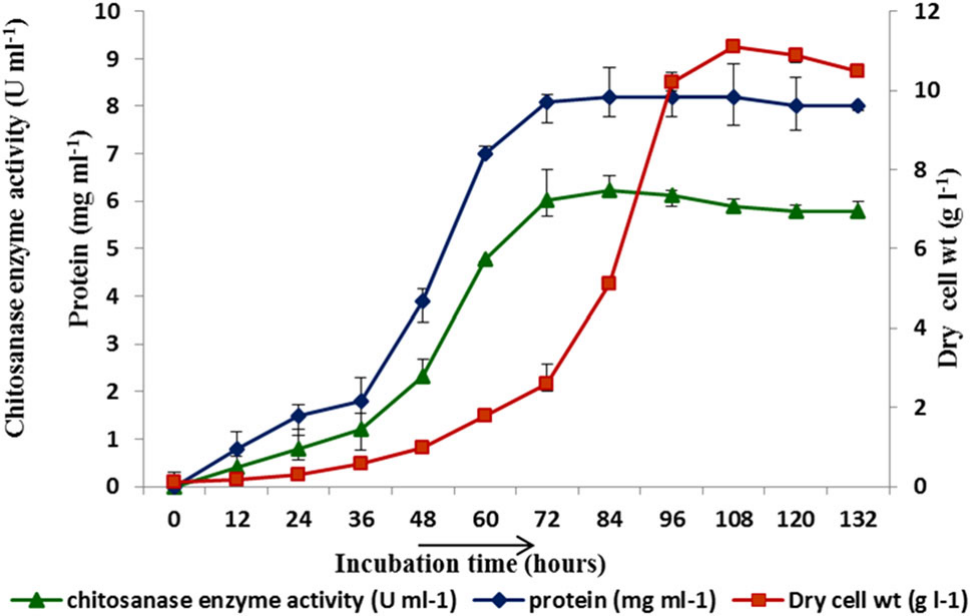
Exochitosanse enzyme production (*triangle*) in U ml^−1^, biomass (*rectangle*) in g l^−1^ and extracellular protein (*diamond*) in mg ml^−1^ with time for *Aspergillus fumigatus* IIT-004

Chitosan-dependent exochitosanase production (6 U ml^−1^) in log phase of growth (Fig. 2) was observed by *Aspergillus fumigatus* in minimal salt medium and there was no enzyme production in dextrose medium. However, in glucosamine minimal salt medium, both growth and exochitosanase production was comparatively lower (4.8 U ml^−1^).

### Purification and characterization

After all the purification steps, 26-fold with 40 % yield were achieved for exochitosanase purification (Table 1). Kinetic parameters, optimum pH, temperature etc. were determined for purified enzyme protein (Table 2). Optimum NaCl concentration was found to be 250 mM for exochitosanase and isoelectric point was found to be 6.5.

**Table 1:** Purification steps of exochitosanase enzyme from *Aspergillus fumigatus* IIT-004

Purification step	Total protein (mg)	Total activity (U)	Specific activity (U mg^−1^)	Purification fold	Yield (%)
Crude broth	10,000	5620	0.5	1	100
Acetone fractionation	5050	4280	0.8	2	70
DEAE Sepharose	1180	3380	3	6	60
CM Sepharose	340	2750	8	16	48
Superdex GL-200	154	1995	13	26	40

**Table 2:** Kinetic parameters of purified exochitosanase from *Aspergillus* sp. when chitosan (>90 % DAC) was used as substrate for enzymatic reaction

Kinetic parameters	Endochitosanase
*K*_m_	8 mg ml^−1^
*V*_max_	5.2 × 10^−6^ IU mg^−1^
*K*_cat_	3 × 10^3^ S^−1^
Activation energy	28 kcal mol^−1^
Optimum pH	5.5
pH stability	7.5
Optimum temperature	40 °C
Temperature stability	Up to 50 °C for 32 h
Isoelectric point	6.5
Molecular weight	64 kDa

Enzyme was inactive against chitin (Table 3) and showed varied activity according to the source of chitosan as only 57 % of enzymatic activity was observed for shrimp shell chitosan (>90 % DAC) as compared to crab shell chitosan (>90 % DAC). Activity decreased with increases in degree of acetylation of chitosan. EDTA, a metal chelator and Zn^2+^ ions improved the enzyme activity to 120 % and was inhibited by Co^2+^ ion (Table 4).

**Table 3:** Substrate specificity of the exochitosanase from *A fumigatus* IIT-004

Substrate	Exochitosanase
Flaked chitin	0
Colloidal chitin	0
Chitosan flakes (shrimp shell, >90 % DAC)	57 ± 2.1
Colloidal chitosan	56 ± 3.2
Chitosan powder (>90 % DAC) (crab shell)	100
Chitosan (>80 % DAC)	68 ± 3.4
Chitosan (>70 % DAC)	43 ± 1.2
Chitosan (>60 % DAC)	21 ± 1.2
Chitosan (>50 % DAC)	9 ± 2.1
Glycol chitosan	12 ± 1.1
Cellulose	0

**Table 4:** Effect of metal ions, organic inhibitors and surfactant on exochitosanase activity

Reagents	Relative activity (%)
	Exochitosanase
Control	100
CaCl_2_	88 ± 2.1
MnCl_2_	98 ± 3.2
HgCl_2_	76 ± 4.1
CuCl_2_	98 ± 2.1
CdCl_2_	90 ± 0.5
MgCl_2_	95 ± 0.4
NiCl_2_	99 ± 0.6
FeCl_2_	87 ± 1.2
ZnCl_2_	102 ± 2.1
BaCl_2_	98 ± 3.1
NiCl_2_	87 ± 1.2
CoCl_2_	32 ± 2.1
EDTA	120 ± 4.3
NBS	100 ± 3.2
DEPC	100 ± 0.2
DTT	90 ± 0.4
β-Mercaptoethanol	100 ± 0.4
IAA	93 ± 4.3
NEM	66 ± 2.1
pCMB	88 ± 2.1
HNB	65 ± 2.1
Tween 20	76 ± 2.1
Triton X-100	88 ± 2.9

### Transglycosylation reaction

**Fig. 3:**
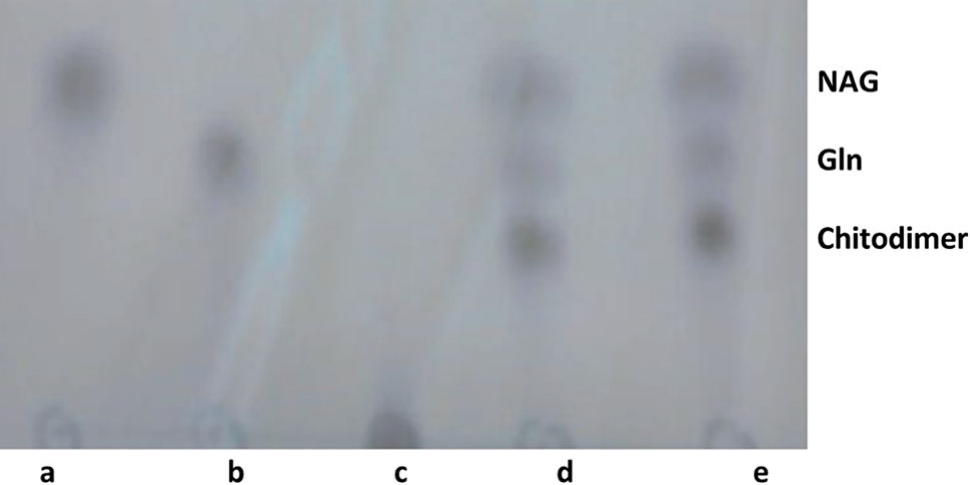
Thin layer chromatography of transglycosylation reaction products of exochitosanase enzyme from *A fumigatus* IIT-004: *a* standard of *N*-acetyl-d-glucosamine; *b* standard of glucosamine; *c* blank; *d*, *e* three bands of COS for *N*-acetyl-d- glucosamine (NAG), glucosamine (Gln) and chitodimer

15 % of ammonium sulfate was found to be optimum for catalyzing transglycosylation reaction. Optimum time and temperature was found to be 36 h and 50 °C respectively. Chitodimer was formed as a result of this reaction (Fig. 3). Other salts used for this study were sodium sulfate and sodium chloride (5–20 %), no such activity was observed with these two salts. Transglycosylation reaction was not observed when only glucosamine or *N*-acetyl-d-glucosamine was used in the reaction mixture.

### Immobilization of chitosanase enzyme

Enzyme was immobilized on PANNFM by adsorption and amidination reaction. Maximum binding was achieved by amidination reaction after 30 min of surface treatment time and 80 % of immobilization efficiency was achieved at 2 h. Protein loading by adsorption and amidination reactions was comparable, but reusability was nil in adsorption process. The value of *K*_m_ for immobilized enzyme was recorded to be 24 mg/ml by Line weaver–Burk Plot. Optimum temperature was found to be 50 °C, optimum pH shifted to 5.8 from 5.5,which could be explained by charge on support material i.e. PANNFM and retained more than 50 % of its catalytic activity even after 10 batches of uses in aqueous solution. Previously reported decrease in enzyme activity after 5 to 6 uses (Kuroiwa et al. 2008) was done on nanoparticles. Storage stability of immobilized also improved as compared to soluble enzyme. More than 60 % of residual activity was observed even after storing more than 30 days at room temperature and minor loss in activity (10–20 %) further even after 30 days of storage.

### Glucosamine and *N*-acetyl-d-glucosamine production

Gln production was achieved by enzymatic hydrolysis of chitosan with free and immobilized enzyme. Maximum gln of 4 mg ml^−1^ could be produced with soluble exochitosanase after 3 h of reaction time. Sodium acetate buffer (200 mM, 5.5) was used in the reaction mixture and chitosan (>90 % DAC, 290 KDa) of 3.5 % (w/v) was found to be maximum substrate concentration. *N*-acetyl-d-glucosamine was also produced by chitosan hydrolysis and concentration of 0.04 mg ml^−1^ was achieved after completing the reaction. Chitosan of (>90 % DAC) was found to be best substrate for glucosamine production.

**Fig. 4:**
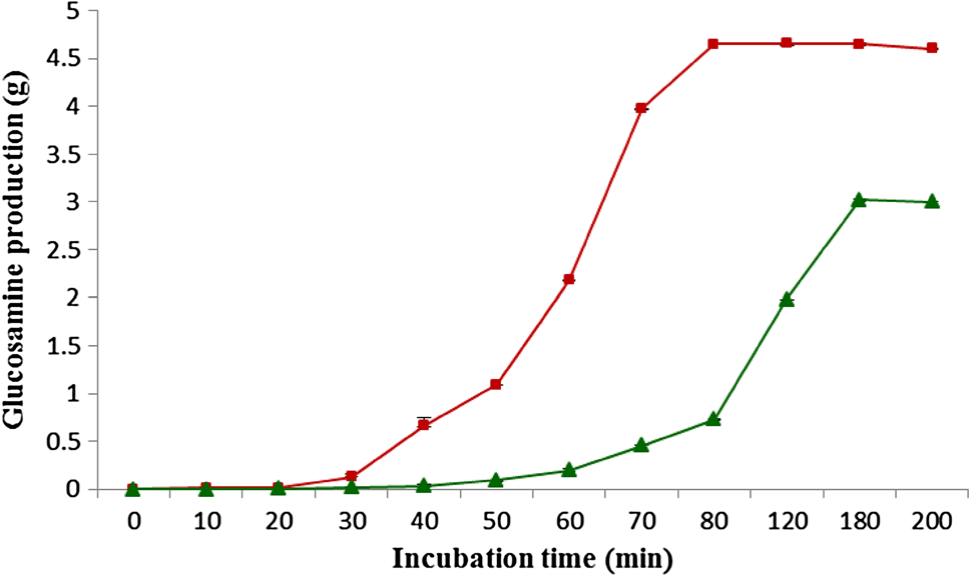
Time course on the production of glucosamine per gram of chitosan by soluble (*square*) and PANNFM-immobilized (*triangle*) exochitosanase: chitosan (>90 % DAC) 1 % (w/v), enzyme: 0.5 g of PANNFM (2.5 mg enzyme g^−1^), sodium acetate buffer (200 mM, 5.5 pH) soluble exochitosanase enzyme (4 U mg^−1^)

Gln production was achieved by immobilized enzyme at 50 °C, and concentration increased up to 70 min. Maximum concentration of 4.6 g was achieved using immobilized exochitosanase enzyme per 10 g of chitosan carrying 1.8 U mg^−1^ specific activities after 2 h of reaction and yield was 80 % as compared to soluble exochitosanase (Fig. 4). Yield of Gln increased with chitosan having lower degree of acetylation and could be produced from a variety of chitosan substrates.

## Discussion

Numerous reports of isolation of endochitosanase secreting microbes from ecological niches are available (Somashekar and Joseph 1996; Thadathil and Velappan 2014); however comparatively, studies on isolation of exochitosanase secreting microbes are scantily available. More number of microbial colonies on colloidal chitosan medium can be explained by the fact that more acetyl groups are removed while preparing the colloidal form of chitosan which improved solubility. Soil collection from fish waste dumping zone also improved the chances of getting diverse group of microbial population as compared to other samples. It was concluded that soil could be good source of novel microbial species secreting industrial enzymes. Enrichment culture technique could be better than direct isolation.

Absence of enzyme production in non chitosan medium indicated that isolate was not producing enzyme constitutively but the enzyme production was induced one.

Optimum temperature of 60 °C have been reported for *Bacillus* sp. strain S65 (Su et al. 2006), *Acinetobacter* CHB101 (Shimosaka et al. 1995), *Bacillus cereus* D-11 (Gao et al. 2008) and *Bacillus cereus* TKU018 (Wang et al. 2009). Chitosanase enzyme from *Paenibacillus* sp. 1794 has been reported to be stable up to 80 °C for 10 min (Zitouni et al. 2013). *Nocardia* and *Aspergillus* sp. have been reported to be stable up to 50 °C (Nanjo et al. 1990; Zhang et al. 2000). Optimum pH of 5.5 has also been recorded for intracellular chitosanase enzyme from *Mucor circinelloides* (Struszczyk et al. 2009), *Nocardia* (Nanjo et al. 1990) and *Aspergillus* sp. (Zhang et al. 2000).

Transglycosylation reaction by enzymes for synthesis of higher oligosaccharides has been studied previously using salts at higher concentration (Usui et al. 1990) and using a microreactor especially designed for lowering the water content (Hsiao et al. 2008). These types of reactions can be enhanced by increasing the substrate concentration, lowering the water content and products are precipitated by reversing the hydrolysis reaction. Similar results have been reported for lysozyme in presence of ammonium sulfate (Akiyama et al. 1995). Change in reaction conditions can lead to unusual catalytic activity, i.e. where hydrolytic enzymes can be used for synthesis of COS by transglycosylation activity.

Lipase immobilization on PANNFM by amidination reaction has been reported previously (Li et al. 2007) which required only 5 min of surface activation. The difference may be attributed to difference in processing and fabrication of nanofibres. Increases in *K*_m_ value indicated low affinity towards substrates which may be attributed to distortion of active sites of enzyme due to covalent attachment or due to reduced access of substrates depending upon mass transfer limitation. Increase in temperature optimum was attributed to restriction of conformational mobility due to covalent bond between matrix and enzyme and as a result higher activation energy, same trend has been observed for immobilized lipase (Li et al. 2007), which could be explained by charge on support material i.e. PANNFM and retention of more than 50 % of its catalytic activity even after 10 batches of uses can be compared to previously reported decrease in enzyme activity after 5 to 6 uses (Kuroiwa et al. 2008) which was done on nanoparticles.

Exochitosanases reportedly cut chitosan and COS from non reducing ends and produce glucosamine (Somashekar and Joseph 1996). Fermentative production of gln has been reported previously (Sitangagng et al. 2010; Jung et al. 2006). Improper mixing of reaction mixture and mass transfer limitation could be explained as reason behind lower yield of product as compared to free enzymes. Immobilized chitosanase have been reported previously for COS production (Kuroiwa et al. 2008; Song et al. 2014; Sinha et al. 2012) but very few reports regarding production of glucosamine are available (Sinha et al. 2011).

Glucosamine can be produced enzymatically for human consumption and with optimization yield can be enhanced at industrial scale for large scale production. Cost effective production can be achieved using chitosan as substrates due to its wider availability as marine waste. Immobilized enzymes are more effective for production of glucosamine due to their high thermo stability and storage stability.
